# People of color’s relative valuing of having therapist performance data inform the referral/assignment process

**DOI:** 10.1080/28324765.2024.2305405

**Published:** 2024-01-22

**Authors:** Michael J. Constantino, Averi N. Gaines, Nicholas J. Hart, James F. Boswell

**Affiliations:** a Department of Psychological and Brain Sciences, University of Massachusetts, Amherst, MA, USA; b Department of Psychology, University at Albany, State University of New York, Albany, NY, USA

**Keywords:** therapist effectiveness data, psychotherapy referrals/case assignment, racial/ethnic mental health care disparities, people of color’s values

## Abstract

Psychotherapists possess measurement-based effectiveness strengths and weaknesses in treating patients with different problems. Moreover, patients report wanting to use these data to optimize their referral/assignment to a therapist. As one way of supporting this value, purposefully matching patients to therapists’ problem-specific strengths has promoted better outcomes than usual case assignment, especially for People of Color (POC). Therapists also possess measurement-based strengths and weaknesses in treating patients with different racial/ethnic identities, and patients report wanting access to this information too. Moreover, naturalistic matching to therapists’ identity-specific strengths has demonstrated preliminary efficacy. Thus, leveraging therapist performance data holds promise for redressing racial/ethnic disparities in psychotherapy. However, additional work is needed to understand the extent to which POC, specifically, view therapist effectiveness data on *what* and *who* they treat as valued inputs into treatment decision-making, including how they compare to other held values. This study used an adapted discounting paradigm to explore such relative valuing among 162 POC. Generally, participants indicated they would sacrifice other common treatment values to see a therapist with a known success rate of approximately 70–80% in treating patients with like problems or identities. The findings help inform how to better serve the needs of historically marginalized individuals.

Mental health care (MHC) therapists can possess, across their average patient, effectiveness strengths and weaknesses (i.e., “performance” differences) depending on the nature of their patients’ presenting problem (Constantino et al., 2021; Kraus et al., 2011, 2016). Such differences are most typically revealed through the within-therapist analysis of “big” data generated by multidimensional, patient-reported outcome measures that assess various mental health concerns. For instance, even when controlling for other patient variables (such as health status and problem severity) known to correlate with treatment outcome, such analysis may reveal that a given therapist is consistently and exceptionally effective at treating their average patient who presents with primary depression or substance misuse (i.e., relative strengths), but reliably ineffective, or perhaps even harmful, when treating their average patient who presents with primary mania or suicidality (i.e., relative weaknesses). In still other problem domains (e.g., anxiety, sleep), this same therapist may have modest effectiveness (i.e., neither a notable strength nor weakness). Once such problem-specific provider effectiveness profiles are established, they could conceivably be shared with patients to assist with a more transparent treatment decision-making process (Constantino et al., 2021; Coyne, 2024).

Notably, diverse MHC patients seem keen on this data-driven idea. For example, a survey of 403 adult community outpatients (51% White, 49% Person of Color [POC^1^]) revealed that greater than 91% of respondents indicated they would use therapist problem-specific performance data when making treatment decisions, if it were made available to them in some digestible manner (Boswell et al., 2021). Moreover, over 90% of these patients also endorsed that (a) it is important to be assigned to therapists *based on* their performance profiles, especially when therapy was previously unsuccessful, and (b) having *access to* and *being matched on* therapist performance data would increase the likelihood of improving through treatment.

A subsequent survey of 308 treatment-familiar (i.e., had prior psychotherapy experience), treatment-engaged (i.e., were currently in psychotherapy), or treatment-seeking (i.e., were currently searching for a therapist) adults^2^ (47% White, 53% POC) generally replicated these results, as participants endorsed a moderate degree of trust in and importance placed on therapist problem-specific performance data (Gaines et al., 2023). They also indicated to a moderate degree the likelihood of referral follow through and confidence in a therapist’s ability to help when aware of provider problem-specific performance data. And extending the Boswell et al. (2021) results, the respondents in the Gaines et al. (2023) study indicated that using therapists’ problem-specific effectiveness data to inform matched referral/case assignment could help somewhat-to-moderately in addressing existing racial/ethnic disparities in the quality of MHC. Thus, overall, these results highlight a patient-valued opportunity to actualize precision care through intentionally matching patients to providers’ unique measurement-based skills and away from their measurement-based shortcomings (in this case with regard to patients’ presenting problems; Constantino & Muir, 2024).

Putting this very idea to the test, a recent double-masked randomized controlled trial of adult community outpatients compared an empirical match algorithm to case assignment as usual (CAU), which is typically based on pragmatic considerations (e.g., wait time, office location) and leaves measurement-based matching to chance (Constantino et al., 2021). Following this manipulated case assignment, treatment itself was delivered fully naturalistically within a large community MHC system. As predicted, and astutely foreshadowed by survey respondents in the aforementioned Boswell et al. (2021) study, matched versus CAU patients had markedly greater symptom reduction and functional improvement across up to 16 weeks of treatment (between-condition *d* = 0.75). Moreover, the benefit of patient-therapist matching was twice as strong for patients who identified as a POC (Boswell et al., 2022), suggesting that leveraging existing therapist performance differences may hold promise for redressing marginalization and other harms that contribute to racial/ethnic disparities in MHC (Alegría et al., 2008; Lê Cook et al., 2014).

Notably, and further implicating racial/ethnic identity as a meaningful contributor to MHC experiences, there is also growing evidence that therapists can possess—much like with diverse problem domains—measurement-based effectiveness strengths and weaknesses depending on the race/ethnicity of the patients they treat (e.g., Coyne, 2024; Hayes et al., 2016; Kivlighan et al., 2019). For instance, whereas a given therapist may be consistently and exceptionally effective when treating their average White patient (i.e., relative strength), they may be ineffective, or perhaps even harmful, when treating their average patient who identifies as a POC (i.e., a relative weakness). Thus, once such provider identity-specific performance profiles are established, they too could conceivably be shared with patients to assist with transparent and personalized decision-making during referral/case assignment (Constantino et al., 2021; Coyne, 2024).

And much like with therapists’ problem-specific performance profiles, diverse MHC patients seem keen on this data-driven idea. For example, in the aforementioned survey of treatment-familiar, treatment-engaged, or treatment-seeking adults (Gaines et al., 2023), respondents endorsed a somewhat-to-moderate degree of trust in and importance placed on therapist identity-specific performance data. They also indicated to a somewhat-to-moderate degree the likelihood of referral follow through and confidence in a therapist’s ability to help when aware of provider identity-specific performance data. Moreover, respondents indicated that using therapists’ identity-specific effectiveness data to inform matched referral/case assignment could help somewhat-to-moderately in addressing existing racial/ethnic disparities in the quality of MHC. Thus, once again, these results highlight a patient-valued opportunity to actualize precision care through intentionally matching patients to providers’ unique measurement-based skills and away from their measurement-based shortcomings (in this case with regard to patient racial/ethnic identities; Coyne, 2024).

In support of this idea, a recent retrospective proof-of-concept study demonstrated that adult community outpatients who were matched by chance to a therapist’s identity-specific strength (prior to naturalistic treatment) had significantly better outcomes than patients who were unmatched by chance (Coyne et al., 2022). Although the benefit of such identity-specific matching awaits replication (including with a prospective experimental design, as was the case for problem-specific matching in Constantino et al., 2021), there is again clear promise for leveraging existing therapist performance differences to redress existing racial/ethnic disparities in MHC. Indeed, one might posit that for patients often marginalized in MHC systems, it could be especially valuable to receive personalized referrals to empirically well-suited therapists based on their problem- and/or identity-specific strengths, especially if they have had poor therapy experiences in the past (e.g., Mays et al., 2017; Sonik et al., 2020).

However, to move beyond such speculation, additional work is needed that prioritizes POC’s perspectives in order to understand more specifically the extent to which they view therapist historical effectiveness data on *what* and *who* they treat as valued inputs into treatment decision-making, including how they compare to other valued therapist factors. Put differently, it is vital not to assume that POC will have the same attitudes as the general population toward using different types of therapist performance information, especially given the unique access and quality disparities they face. Supporting this point, POC have endorsed experiencing a greater sense than their White counterparts that past difficulties finding a therapist were attributable to their racial/ethnic identity (Gaines et al., 2023). Additionally, prior work has uncovered meaningful differences between POC and White individuals on their preferences for/relative valuing of various treatment and therapist characteristics (e.g., therapist multicultural training/experience, therapist use of culturally adapted treatments, patient-therapist racial/ethnic identity match; Castro et al., 2015; Ellis et al., 2019; Swift et al., 2015).

Nonetheless, no prior research has expressly asked POC about their attitudes toward and valuing of using therapist problem- and identity-specific performance data for MHC referral/assignment, including how the personal utility of these data would compare to other factors that patients often value (e.g., contained treatment cost, therapists possessing preferred demographic characteristics). To address this gap, the present study drew on an adapted discounting paradigm that has been increasingly applied to psychotherapy topics (e.g., Boswell et al., 2018; Swift et al., 2015). Discounting data can provide information regarding what overall caseload-level of effectiveness a therapist would need to demonstrate (i.e., their overall good-outcome success rate when treating their patients with a specific presenting problem or racial/ethnic identity) for a POC to willingly discount (i.e., sacrifice) other common therapist-related preferences/values in favor of using such therapist performance information in the referral/assignment process. Put differently, discounting data in this case can provide a preliminary picture of how POC weight various MHC-related values, which can be a valuable input into patient-centered care.

For our first aim, we separately explored descriptive data for these discount levels (relative to each of eight common therapist-related preferences/values) for both therapist problem- and identity-specific performance information. For our second aim, we examined whether POC’s discount levels differed significantly for any common therapist-related preferences/values between the two types of therapist performance data. These findings can help provide MHC therapists and systems with preliminary guidelines for when and how to make referrals/assignments in a manner that is personally responsive to patients’ valued preferences (an evidence-based principle of change; Swift et al., 2018).

## Method

### Dataset overview

Data for the present study were collected as part of the same MTurk-based human intelligence task (HIT) that generated the aforementioned Gaines et al. (2023) survey data. The present subsample included only those respondents who identified as a POC (operationalized as anyone who endorsed a non-White and/or Hispanic, Latino, Latina, or Latinx identity). Importantly, after excluding participants because of invalid data (as discussed further in the subsequent data analysis and results sections), the remaining effective sample included the exact same POC that were represented in the Gaines et al. (2023) study. Broadly, the MTurk platform allows adult “workers” to complete HITs, including psychological surveys, for monetary compensation. The suitability of the MTurk population to the current study is underscored by 36% of workers identifying as a POC (Buhrmester et al., 2011). Moreover, MTurk workers report mental health concerns at a level comparable to and in some cases higher than the general population (Shapiro et al., 2013); thus, as noted, a meaningful subset of individuals have likely interacted with (or plan to interact with) MHC therapists in some capacity. Finally, MTurk workers are more demographically comparable to the broader U.S. population than are other Internet or undergraduate student samples (Buhrmester et al., 2011).

### Participants

Study participants were 253 adults aged 18 or older who (a) identified as a POC (including multiracial individuals who identified as partly White); (b) were current US residents; (c) had previous experience with, were currently engaged in, or were actively seeking psychotherapy; and (d) had completed at least 50 prior MTurk HITs, had a 95% approval rate across those HITs, and had a designation as “MTurk Master” (indicating they had a track record of providing valid data).

### Measures

Overlapping with the Gaines et al. (2023) study, participants initially reported on their demographic and clinical characteristics. The demographic items included: age, gender, sexual orientation, race/ethnicity, marital status, annual income, religious affiliation, and education. On a scale from 0 (*not at all*) to 4 (*very*), participants also indicated their strength of racial/ethnic identity and strength of religious affiliation. The clinical items assessed experience of various mental health concerns and treatment modalities, as they specifically related to the respondent’s previous engagement with, current engagement with, and/or active search for MHC. Also overlapping with the Gaines et al. (2023) study, participants completed two attention check questions that served as validity checks. As an additional validity check at the end of the survey, participants self-reported on the quality of their data.

Unique to the present study, participants next completed a series of choice tasks informed by a delay-discounting paradigm (Critchfield & Kollins, 2001) to assess the valuing of using therapist effectiveness data to inform the referral/case-assignment process relative to the following eight therapist factors that MHC patients often value: (1) has many years of experience, (2) has a small co-pay (i.e., a flat fee one pays for a health service beyond what is covered by their insurance), (3) works at a convenient location, (4) has a shared gender identity, (5) has a shared sexual orientation, (6) has a shared racial/ethnic identity, (7) administers a patient-preferred treatment type, and (8) comes recommended by a friend or family member. In each case, participants rated their preference for experiencing the presumed valued treatment factor versus discounting (i.e., sacrificing) that factor to work with a therapist who possesses a certain known historical caseload-level of effectiveness in treating people with a similar presenting problem and then again in treating people with a similar racial/ethnic identity (16 items total). Here caseload-level effectiveness referred to a percentage of the hypothetical therapist’s patients who improved significantly (e.g., a 50% success rate, meaning that 5 out of every 10 of their patients who had a certain type of presenting problem demonstrated significant improvement after working with this therapist).

**Figure 1.**
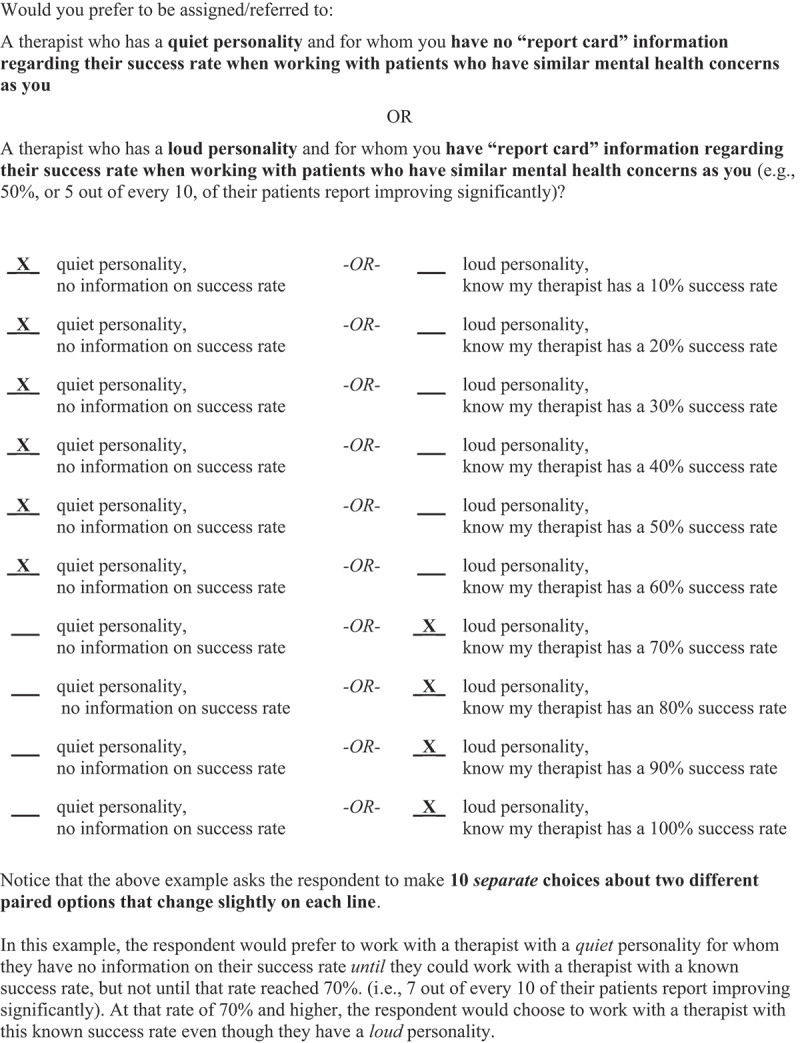
Example discounting item.

To socialize participants to this task, they were presented with the example item depicted in Figure 1. Here the presumed preference/value is having a therapist with a *quiet* personality, though to experience this value would also mean having no information on the therapist’s historical caseload-level effectiveness in treating patients with a similar mental health concern. This value is compared to a therapist with a presumed less valued *loud* personality, though to experience this characteristic would also mean possessing information on the therapist’s historical caseload-level effectiveness in treating patients with a similar mental health concern; that is, the participant would know 10 such success rates, ranging from 10–100%. Working their way down the comparator levels, this example shows that the hypothetical respondent would prefer to work with a quiet-personality therapist for whom they have no success data *until* a loud-personality therapist had a known 70% historical success rate at treating their problem; that is, at this level or higher, the respondent would weight favorably being assigned/referred to the empirically effective therapist and endure the less desired therapist characteristic of being loud. This cross-over juncture of 70% is referred to as the *indifference point* (IP)—again, the therapist success level at which one is no longer indifferent to the benefit of this new characteristic, which would require giving up the old, presumably positive characteristic. To reiterate, for each of the eight values examined in the present study, we repeated the questions for therapist success rates in treating people with a similar problem and then in treating people with a similar racial/ethnic identity.

### Procedure

As described in Gaines et al. (2023), data were collected between October 2021 and June 2022. Specifically, we recruited participants through the MTurk crowd-sourcing platform, intentionally sampling in the first wave of data collection POC who met the previously stated inclusion criteria. Following their acceptance of the HIT, participants were directed to Qualtrics to complete the consent form and survey (part of which included the present discounting tasks relevant to the present study). As also noted in Gaines et al. (2023) patients were compensated $5 for the 25-minute survey, which is at the upper end of standard industry rates. The institutional review board at the first author’s university approved the study protocol (no. 2931).

### Data analysis

As described in Gaines et al. (2023), we first examined the validity of participant responses and excluded in full anyone who did not endorse being treatment-familiar, treatment-engaged, or treatment-seeking; endorsed every or not any racial/ethnic identities; failed to complete the full survey; failed either of the attention check questions; completed the survey more than once (as per a duplicate IP address); completed the survey in an unrealistically brief amount of time (i.e., fewer than 8 minutes, based on piloting with undergraduate research assistants who were instructed to complete the survey as quickly as possible while still accurately processing the instructions); indicated we should not use their data due to poor self-reported quality; and/or demonstrated invalid response patterns (as determined by trained research assistants) on greater than 90% of the discounting tasks. (Note that for participants who had invalid responses on fewer than 90% of the tasks, we retained their data for the valid items only). Consistent with previously published MTurk studies in psychotherapy research (e.g., Bernecker et al., 2017; Constantino et al., 2019; Swift et al., 2015), we expected to exclude approximately 30% of the respondents because of invalid data.

Next, as also described in Gaines et al. (2023), we conducted descriptive analyses on participants’ demographic and clinical characteristics. Finally, for our primary analyses, we followed the procedures from prior adapted discounting studies in psychotherapy research (e.g., Boswell et al., 2018; Swift et al., 2015) by calculating IPs (viz. the average point at which one factor is first preferred over another, as per the comparisons described previously). For the present study’s descriptive first aim, the mean IPs signified the average therapist success rate at which a participant would first sacrifice a particular (and commonly held) treatment preference/value being met in order to see a therapist with that known success rate. For the present study’s inferential second aim, we used paired sample *t*-tests for each of the eight common preferences/values to examine differences in the average IP between therapist effectiveness profile type (i.e., problem-specific vs. identity-specific). We also calculated Cohen’s *d* as a measure of effect size for these differences.

## Results

### Preliminary

As described in Gaines et al. (2023), we excluded 91 (36%) participants from the POC subsample because of invalid data. Thus, fully consistent with Gaines et al. (2023) our effective sample was 162 participants who identified as a POC. As noted previously, the present exclusion percentage is consistent with prior MTurk studies on psychotherapy topics. As the invalid data were largely a function of carelessness in responding, it is unlikely there were systematic psychological differences between those whose data were versus were not included in the main analyses.

### Demographic and clinical characteristics

Tables 1 and 2 present the effective sample’s demographic and clinical characteristics, respectively.

**Table 1.** Participant demographic characteristics (*N* = 162)

Variable		*M*	*SD*	*n*	%
Age (years)		38.36	9.58	162	
Gender					
	Genderqueer/nonbinary			4	2.47
	Cisgender man			77	47.53
	Cisgender woman			75	46.30
	Transgender woman			1	0.62
	I prefer to not answer this question			5	3.09
Sexual orientation					
	Asexual			2	1.23
	Bisexual			24	14.81
	Gay			6	3.70
	Heterosexual or straight			117	72.22
	Lesbian			5	3.09
	Pansexual			3	1.85
	Queer			2	1.23
	I prefer to not answer this question			3	1.85
Race(s)/Ethnicity(ies)^a^					
	American Indian or Alaska Native			15	9.26
	Black or African American			67	41.36
	East Asian			18	11.11
	Hispanic, Latino, Latina, or Latinx			45	27.78
	Middle Eastern or North African			7	4.32
	Native Hawaiian or Other Pacific Islander			4	2.47
	South Asian			6	3.70
	Southeast Asian			10	6.17
	White			45	27.78
Marital Status					
	Divorced			13	8.02
	Married or committed partnership			70	43.21
	Single, never married			73	45.06
	Separated			1	0.62
	Widowed			4	2.47
	I prefer not to answer this question			1	0.62
Annual household income					
	Less than $20,000			10	6.17
	$20,000 to $39,999			39	24.07
	$40,000 to $59,999			38	23.46
	$60,000 to $79,999			30	18.52
	$80,000 to $99,999			20	12.35
	$100,000 to $119,999			8	4.94
	$120,000 to $139,999			4	2.47
	$140,000 to $159,999			2	1.23
	More than $160,000			8	4.94
	I prefer to not answer this question			3	1.85
Religious affiliation					
	I have a religious affiliation			60	37.04
	I do not have a religious affiliation			95	58.64
	I prefer to not answer this question			7	4.32
Highest level of education					
	Did not complete high school or GED			1	0.62
	Completed high school or GED			21	12.96
	Some college			32	19.75
	Two-year college degree			21	12.96
	Four-year college degree			66	40.74
	Some graduate or medical school			5	3.09
	Completed a master’s degree			11	6.79
	Completed a doctoral or MD degree			4	2.47
	I prefer to not answer this question			1	0.62
Strength of racial/ethnic identity^b^		3.47	0.77	159	
Strength of religious affiliation^b^		2.95	0.96	59	

*Note. M* = mean; *SD* = standard deviation.

^*a*^Categories sum to greater than 162 because participants were permitted to endorse more than one racial/ethnic identity.

^a^Item was assessed using a Likert scale of 0 (*not at all*) to 4 (*very*).

**Table 2.** Participant clinical characteristics (*N* = 162)

	Past Treatment		Current Treatment		Treatment Sought	
Item	*n*	%	*n*	%	*n*	%
*Mental health concern*						
Anxiety	82	50.62	55	33.95	60	37.04
Attentional problems	13	8.02	8	4.94	14	8.64
Depression	85	52.47	56	34.57	54	33.33
Developmental disability	1	0.62	3	1.85	5	3.09
Eating disorder	6	3.70	4	2.47	6	3.70
Learning disability	2	1.23	3	1.85	6	3.70
Mania	4	2.47	2	1.23	3	1.85
Psychotic symptoms	5	3.09	3	1.85	4	2.47
Quality of life	15	9.26	14	8.64	15	9.26
Self-harm	10	6.17	5	3.09	4	2.47
Sexual functioning	4	2.47	2	1.23	4	2.47
Social conflict	9	5.56	5	3.09	7	4.32
Sleep functioning	10	6.17	13	8.02	11	6.79
Substance use	14	8.64	9	5.56	4	2.47
Suicidal thoughts	17	10.49	5	3.09	3	1.85
Trauma	17	10.49	11	6.79	17	10.49
Violence	0	0.00	0	0.00	2	1.23
Work functioning	3	1.85	1	0.62	5	3.09
Another option not listed	5	3.09	1	0.62	2	1.23
*Treatment modality*						
Individual therapy	114	70.37	73	45.06	84	51.85
Group therapy	22	13.58	8	4.94	7	4.32
Couples therapy	8	4.94	4	2.47	6	3.70
Family therapy	13	8.02	5	3.09	6	3.70
Case management	9	5.56	6	3.70	7	4.32
Hospitalization	12	7.41	2	1.23	4	2.47
Partial hospitalization	8	4.94	3	1.85	4	2.47
School-based services	5	3.09	2	1.23	4	2.47
Medication	42	25.93	32	19.75	22	13.58
Another option not listed	1	0.62	0	0.00	1	0.62

*Note*. Categories sum to greater than 162 because participants were permitted to endorse more than one option.

### Therapist problem-specific caseload-level effectiveness

Mean IPs for the eight therapist problem-specific effectiveness discounting tasks are presented in the first column of Table 3. The presumed preferences/values are listed in order from the one it would have taken the lowest level of known therapist caseload-level effectiveness (i.e., success rate) to sacrifice to the one it would have taken the highest level. Broadly, no respondent indicated they would sacrifice any current preference/value to work with a particular therapist *until* they knew that therapist had close to a 70% effectiveness success rate in treating their problem; in other words, their track record indicated being noticeably better than chance at helping their average patient with a similar issue (a genuine relative strength). Zooming in, it would have taken between approximately 68–73% of known therapist caseload-level effectiveness, on average, for the participants to sacrifice various demographic identity matches, and the therapist being recommended by a friend or family member, having a lot of experience, and working at a convenient location. However, POC held tighter to the values of receiving a preferred treatment type and having a smaller co-pay; that is, before they would have sacrificed these features, they would have needed to know a therapist was historically successful in treating more than 8 of every 10 patients with a similar problem.

**Table 3.** Indifference points for therapist problem-specific vs. Racial/ethnic identity-specific effectiveness data (*N* = 162)

	Problem-Specific Indifference Point %			Identity-Specific Indifference Point %			Group Difference			
Preference	*M*	*SD*	*n*	*M*	*SD*	*n*	*t*	*df*	*p*	*d*
Shared sexual orientation	68.01	21.24	161	69.63	20.64	161	−1.50	160	.135	0.12
Recommended by a friend or family member	69.50	16.80	161	71.24	17.20	161	−1.50	160	.135	0.12
Shared gender identity	69.81	23.47	161	69.69	22.40	161	0.09	160	.931	0.01
Shared racial/ethnic identity(ies)	70.81	19.00	161	70.25	19.14	161	0.46	160	.644	0.04
Many years of experience	71.38	15.28	160	69.50	17.30	160	1.92	159	.057	0.15
Convenient location	72.88	16.99	160	71.75	18.91	160	0.98	159	.328	0.08
Preferred treatment type	81.54	16.85	162	78.33	18.46	162	2.62	161	.010	0.21
Small co-pay	83.38	16.38	160	80.06	19.54	160	2.94	159	.004	0.23

*Note. M* = mean; *SD* = standard deviation. Some *ns* are smaller than 162 due to missing data. The indifference point indicates the average effectiveness rate at which participants discounted being referred to a therapist who met a commonly held preference in favor of seeing a therapist with XX% problem-specific or identity-specific effectiveness rate.

### Therapist identity-specific caseload-level effectiveness

Mean IPs for the eight therapist identity-specific effectiveness discounting tasks are presented in the second column of Table 3. The same high-level pattern emerged—respondents would not have sacrificed any preference/value until they knew that therapist had close to a 70% success rate across their caseload in treating people who possessed the same racial/ethnic identity as the respondent (a genuine relative strength). Moreover, the same preferences/values emerged for which it would have taken approximately 69–72% of known therapist caseload-level identity effectiveness (i.e., various demographic identity matches, and the therapist being recommended by a friend or family member, having a lot of experience, and working at a convenient location) and for which it would have taken close to an 80% success rate (i.e., receiving a preferred treatment type and having a small co-pay).

### Comparing therapist problem- versus identity-specific effectiveness

Comparisons between IPs for therapist problem-specific and identity-specific effectiveness discounting tasks are presented in the third column of Table 3. Notably, the amount of therapist caseload-level effectiveness it would have taken to sacrifice receiving a preferred treatment type or having a small co-pay was lower for identity- versus problem-specific effectiveness (though note that the effect sizes were both small). For no other presumed preference/value was there a significant difference on IPs when comparing the two types of therapist caseload-level effectiveness.

## Discussion

This study provided an initial view into POC’s relative valuing of one way to use therapist data for more personalized care—that is, being made aware of and choosing to see a therapist based on their measurement-based strengths, even if it comes at the expense of other presumed preference/values. Overall, the data indicated a somewhat intuitive point at which knowing and using a therapist’s historical caseload-level effectiveness information would become more preferred/valued than experiencing other provider characteristics about which patients often care; that is, when the therapist’s success rate in treating a patient with a certain problem or a certain racial/ethnic identity was more than a 50/50 proposition. Roughly speaking, it would have taken a therapist having at least a 70% success rate for POC to sacrifice most other presumed preferences/values (and closer to 80% to sacrifice receiving one’s preferred treatment and having a small co-pay). However, for these two characteristics that POC appeared to hold onto more tightly, the “crossover” point was a bit lower when they knew therapists had empirically determined identity- versus problem-specific strengths. Although this difference could suggest a stronger valuing of making actionable therapist identity- versus problem-specific effectiveness data among POC, with the IPs only differing only by about 3%, it is currently unclear if these statistically significant differences are clinically meaningful.

The implications of the present results can be considered at multiple levels. At the most basic *patient* level, the results give a sense, preliminarily at least, of how POC weight therapist-related preferences/values. Most centrally, they tell us there may be specific thresholds of therapist caseload-level effectiveness that would render such information a personally key determinant of who they would prefer to see for therapy—even if they needed to sacrifice some other valued feature of their provider to do so. Thus, at the pertinent threshold, attempts to make referrals/assignments based on therapists’ historical success rates in treating certain problems or people might now be considered (again, preliminarily) *patient-centered* actions, as opposed to actions that are imposed by a care system, clinician, or researcher without knowing how they align with what patients (in this case, POC) most want and need.

Relatedly, then, the evidence-based beneficial action of prospectively matching patients to therapists’ measurement-based, problem-specific strengths (Constantino et al., 2021), which was especially strong for POC (Boswell et al., 2022), aligns well with POC’s therapist-related preferences/values; in other words, such matching would be evidence-based *and* patient-centered in nature. Thus, at the *therapist* level MHC clinicians may be wise to track their multidimensional outcomes, establish their performance profiles, and transparently reveal them to help POC make informed treatment decisions (Coyne, 2024; Muir et al., 2019). Arguably, these actions take on urgent significance when considering that therapists are largely inaccurate in assessing their own measurement-based, problem-specific strengths and weaknesses (Constantino et al., 2023). Then, when possible, it may be that therapists should try to coordinate care so they prioritize seeing POC who have the types of problems they excel at treating (based on measurement, not self-perceptions) while avoiding working with POC who have the sorts of problems they treat less successfully (or for which they may even be harmful).

The present data add complexity, though, to the evidence-based action of matching. To be truly evidence-based *and* patient-centered, therapists’ past excellence may only “matter” to a POC when it is high enough (e.g., over a 70% success rate) to precipitate their relative valuing and weighting of this therapist characteristic over others. With this example, a therapist who established a 60% success rate in treating depression (i.e., 6 of every 10 their patients with this primary concern showed significant improvement) may not be a great value-aligned match for a patient who holds a strong preference for another factor they do not meet (such as a shared gender identity). Of course, there could also be a scenario in which the therapist does match on gender identity, but has only a 20% success rate. Although the patient may not value this information to the point they would sacrifice the gender match, you could still argue that it would be beneficial not to meet this patient’s gender-match preference given it would also result in them seeing a known *ineffective* therapist. To be sure, more research is needed to know precisely when to meet which values over others, and if and when an evidence-based practice like matching should potentially override other held values to the extent it can help address the disparities in MHC access, engagement, and quality that POC often face (even if it creates some tension with one’s value system). Notably, such tension was not present in the Constantino et al. (2021) match trial, as both patients and therapists were masked as to whether they were matched or not. Thus, even to matched patients, they may have believed they were assigned to a therapist based on other preferences/values that were considered in the usual assignment methods (e.g., a convenient office location).

Similar to problem-specific effectiveness, the evidence-based action of matching patients to therapists’ historical and measurement-based identity-specific strengths (Coyne et al., 2022) aligns well with POC’s therapist-related preferences/values; in other words, such matching would also be both evidence-based *and* patient-centered. Thus, again at the therapist level, MHC clinicians may also be wise to track their outcomes by the racial/ethnic identities of the patients they treat. They can then establish their complementary identity-specific performance profiles and transparently advertise them to help POC make informed treatment decisions with even more therapist data in hand (Coyne, 2024). Then, when possible, another way therapists can coordinate care is by treating the patients who have identities that match their measurement-based strengths and refer out patients who have identities that align with their measurement-based weaknesses. As noted with problem-specific strengths, though, the meeting of values is complex, especially when a therapist’s historical success rate of working with patients of certain identities does not meet the threshold of becoming more valued than something else. As noted previously, future research is needed to better understand the complexities of integrating—versus if/when to potentially override—evidence-based practices and patient-valued care.

Importantly, the existing work on measurement-based matching to therapists’ identity-specific strengths (as one means to actualizing a patient’s value for using provider performance data) is limited in several important regards. First, Coyne et al. (2022) tested the match effect in archived data for which they retrospectively identified patients who had been matched in this manner purely by chance. Thus, future research is needed to test such identity-specific matching prospectively, as was the case in the Constantino et al. (2021) trial that centered on therapists’ measurement-based, problem-specific strengths. Second, in both the Coyne et al. study and the present study, identity-specific strengths only crudely reflected therapist effectiveness differences in treating any POC versus White patients. Future work is needed that examines within-therapist effectiveness differences more granularly within the overarching domain of treating POC (e.g., some therapists could have a relative strength in treating their average Black patient, but a relative weaknesses in treating their average American Indian patient).

Finally, the present valuing data could also, preliminarily at least, inform *systems*-level actions toward personalized care for POC. For instance, if in a care network there is a therapist with at least a 70% success rate in treating the problem and/or racial/ethnic identity a new patient has, then intake workers can intentionally make this patient-valued assignment. However, if such a therapist does not currently exist within or even feasibly outside of the network, then the present findings would suggest that intake workers should try to meet one or more other preferences/values the patient possesses (e.g., seeing a therapist who simply *shares* a salient identity, such as race/ethnicity). Importantly, this would still be a form of evidence-informed personalization by way of meeting preferences (Swift et al., 2018).

Again, though, such actions are complex. If a therapist with a 70% success rate does not exist and the system is purposefully meeting a different patient-centered value, it may still be important to use any existing data on therapists’ historical caseload-level effectiveness. For example, consider if there were two therapists who share a racial/ethnic identity with the given new patient, with one having a 50% success rate and other a 10% success rate in treating their patients with a similar problem and/or racial/ethnic identity. In this hypothetical scenario, it may be important to assign this case to the 50% effective clinician. This assignment would still meet the shared identity value, but it would also prioritize the therapist who is at least average (i.e., helps 5 of every 10 of their patients with a similar problem and/or racial/ethnic identity improve significantly) versus notably ineffective or even harmful (i.e., helps only 1 of every 10 of their patients with a similar problem and/or racial/ethnic identity improve significantly). Although the patient may not have valued the effectiveness data all that much in this scenario (at least not over the shared identity value), it does not mean the care system should ignore poor-performing clinicians on a short-list of otherwise value-matched clinicians for a given patient. Indeed, the present discounting task was not oriented toward the valuing of low therapist success rates and the threshold for which it would be so low that it would override a preference to see a given clinician even if they possessed the other preferred value. Future discounting research could engage this orientation as a complement to the present study.

Notably, should future research show different IPs than we found, either with POC in general or other more finely distinguished subsamples, a care system’s ability to meet the value of seeing a therapist with a certain historical success rate would be affected. For example, a crossover point of 40% (vs. the approximately 70% we saw for most therapist characteristics) would be an easier threshold for a system to meet; that is, if the average patient would sacrifice other values to know that a given therapist with whom they may work had at least a 40% success rate in treating certain people or problems, more of those therapists would exist within a given system, which would allow for more readily meeting this value (e.g., through explicit matching). In this case, the patient-centered value would likely have represented knowing concretely and transparently a therapist’s effectiveness track record over not knowing and preserving other values. Moreover, it would also have meant that a patient did not necessarily need the track record to show high levels of excellence, but rather only some effectiveness (which, again, would allow systems to meet this value more expediently).

However, if the crossover point was higher—for example, only at 90% effectiveness would a patient prioritize a therapist’s historical success rate over other values—then systems would have a harder time meeting this value because they may not have an abundance of such therapists to which to prospectively match the patient. In the absence of such options, the evidence-based and patient-centered actions may be to preserve other preferred factors, such as lower co-pay, etc., while, as noted previously, still assigning away from a very ineffective therapist, if possible. Finally, should future research show bigger differences in valuing between therapists’ identity- and problem-specific effectiveness information than we saw presently, whether with POC or other subgroups, it may speak to the importance of prioritizing a certain type of therapist effectiveness data in clinical decision-making (in other words, it may be that not all such data are created equally for all people).

Of course, the present results and current implications should be considered with the study-specific limitations in mind. First, as noted, we examined relative valuing *across* different underrepresented racial/ethnic identities, which neglected heterogeneity among POC. Second, although having to exclude a fair number of respondents due to invalid data and careless responding is common with MTurk studies, it is nevertheless unclear whether the respondents whose data were versus were not included in our analyses may have differed systematically in some way, thereby creating a potential unknown bias in our results. Third, although MTurk samples are representative of the US population on key demographic and clinical characteristics, it remains that the present results may not generalize beyond a sample of treatment-familiar, treatment-engaged, or treatment-seeking individuals who responded to this survey via this specific online platform. Fourth, although our sample was recruited based on being actual past, present, or future MHC patients, we appreciate that the recruitment setting was not the same as sampling directly from MHC service agencies. Thus, future work is needed to replicate the present findings with patients presenting to a specific service for an intake (i.e., an immediate decision-making inflection point).

Fifth, for this preliminary snapshot, we examined just one version of relative valuing—i.e., fully sacrificing a current preference/value to experience something else. Although this provides some sense of the relative strength of one’s preferences/values, in an ideal world people would not have to choose so discretely. In fact, given that meeting patient preferences is an important transtheoretical and transdiagnostic principle of change (Swift et al., 2018), systems would be wise to meet as many preferences/values as possible in order to make personalized care even more potent. Sixth, for this initial study, and akin to a direct effect, we did not calibrate respondents’ valuing to their perceptions of an average success rate of therapists in general. That being said, and akin to an interactive effect, it is possible that valuing thresholds can differ systematically based on such general perceptions or expectations. Unfortunately, we did not assess this moderating variable; thus, it could become the focus of replication and extension. Finally, using deciles to assess IPs limited precision. For example, when a participant crossed over at 70% effectiveness, it is unclear if this would have more precisely occurred at 61% or closer to 69%. Future work could make use of visual analogue scales to increase the precision of relative valuing.

Limitations aside, the present findings can help the field combat uniformity myths and responsively tailor therapist performance data to cultural context. When considered alongside Gaines et al. (2023) finding that POC versus White stakeholders endorsed significantly more positive attitudes toward harnessing therapist data, the MHC field has an emerging and promising means for doing better by historically disadvantaged and underserved individuals.

## Data Availability

The data that support the findings of this study are available from the corresponding author upon reasonable request.

## Notes

1. Hereafter, we use POC as the abbreviation for both *Person* of Color and *People* of Color.

2. These respondents were recruited via Amazon Mechanical Turk (MTurk), an online data collection platform. Notably, MTurk participants have reported mental health difficulties at a rate commensurate with the general population (Shapiro et al., 2013). Thus, a meaningful subset have likely interacted with (or intend to interact with) MHC therapists, which rendered them suitable for the Gaines et al. (2023) study.
